# AD7c-NTP Impairs Adult Striatal Neurogenesis by Affecting the Biological Function of MeCP2 in APP/PSl Transgenic Mouse Model of Alzheimer's Disease

**DOI:** 10.3389/fnagi.2020.616614

**Published:** 2021-01-20

**Authors:** Pan Li, Wei Quan, Zengguang Wang, Yuan Chen, Huihong Zhang, Yuying Zhou

**Affiliations:** ^1^Department of Neurology, Tianjin Huanhu Hospital Affiliated to Nankai University, Tianjin, China; ^2^Tianjin Key Laboratory of Cerebral Vascular and Neurodegenerative Diseases, Tianjin Neurosurgery Institute, Tianjin Huanhu Hospital Affiliated to Nankai University, Tianjin, China; ^3^Department of Neurosurgery, General Hospital of Tianjin Medical University, Tianjin, China; ^4^Tianjin Key Laboratory of Injuries, Variations and Regeneration of Nervous System, Tianjin Neurological Institute, Tianjin, China

**Keywords:** Alzheimer disease, AD7c-NTP, neurogeneis, neural plasticity, DNA methylation, methyl-CpG binding protein 2, protein phosphorylation, epigentics

## Abstract

The processes by which neural stem cells (NSCs) and neural precursor cells (NPCs) transform into the characteristic lineages observed in Alzheimer's disease (AD) are poorly characterized. Understanding these processes is of critical importance due to the increased prevalence of AD and the lack of effective AD strategies. Here, we used immunohistochemistry and Western blot to find out if MeCP2 was phosphorylated at a specific amino acid residue, Serine 421 (S421), and activated in response to AD-induced damage in amyloid precursor protein (APP)/PSl transgenic mice, altering its nuclear to cytoplasmic shuttling. Epigenetic examinations combined with chromatin immunoprecipitation and methylated DNA immunoprecipitation revealed that the translocation of MeCP2 from the nucleus to cytoplasm led to the loss of lineage-specific gene promoters (such as *Gfap, Nestin*, and *Dcx*), decreased transcriptional repression, and the activation of gene expression. Immunofluorescence data demonstrated that neurogenic progenitors with high levels of active phosphorylated MeCP2 at S421 (MeCP2 pS421) possessed a high probability of development into doublecortin (DCX)-expressing cells. AD7c-NTP will control neurogenic progenitor regeneration through its effects on MeCP2 pS421, leading to altered lineage-specific gene expression. This adds to the growing list of biological effects of AD7c-NTP in the brain and highlights MeCP2 as relevant to the plasticity of neural cells in the AD mice striatum.

## Introduction

AD is the major form of senile dementia, a debilitating neuronal disorder that impairs memory, language, executive functions, and visuospatial skills (Dubois et al., 2014). AD is characterized by amyloid deposits, neurofibrillary tangles, and a loss of neuronal cell viability around the forebrain, cortex, and limbic system (Horgusluoglu et al., 2017). During development, neurogenic progenitors become restricted to the subependymal ventricular zone (SVZ) (Lois and Alvarez-Buylla, 1993; Lim et al., 1997) and the subgranular layer (SGL) of the dentate gyrus (Ihunwo et al., 2016; Paredes et al., 2018), leading to impaired neurogenesis and inhibited cognitive functions in the elderly. However, in the context of AD (Jin et al., 2004a,b; Lopez-Toledano and Shelanski, 2004, 2007; Boekhoorn et al., 2006; Herran et al., 2015), transgenic mice harboring ≥3 AD-linked amyloid precursor protein (APP) mutations show higher levels of proliferation and neuronal differentiation (Jin et al., 2004a,b; Lopez-Toledano and Shelanski, 2004, 2007; Herran et al., 2015). Increased Ki-67 staining is also observed (Boekhoorn et al., 2006). Therefore, the activation, regulation, and related molecular mechanisms of neurogenesis after AD injury will become the therapeutic intervention target.

The neural thread protein (NTP) is overexpressed in AD lesions and contributes to disease pathogenesis (De la Monte and Wands, 1992). The AD-associated neural thread protein (AD7c-NTP) was first cloned (De La Monte et al., 1996) and found to be dysregulated in neuronal axons. It has since been shown to codistribute with neurofibrillary tangles (NFTs) and positively correlates with NFTs (Monte et al., 1997). Enhanced AD7c-NTP expression occurs during early AD, prior to NFTs formation, and its function is related to neuritis germination and cell death (Monte et al., 1997), suggesting its association with AD neurodegeneration (Kahle et al., 2000; De la Monte and Wands, 2001a,c). All of the above findings have raised the key questions regarding the ability of AD7c-NTP to affect the neurogenesis in the brain following AD injury, as well as molecular pathways underlying the proliferation and differentiation of NSCs and NPCs.

DNA methylation at cytosine residues in the CpG islands regulate the plasticity of neural cells (Kohyama et al., 2008; Desai et al., 2019) by suppressing expression of key genes. Gene methylation is essential to fetal development, the remodeling of tissue, regulated proliferation, and cell differentiation (Li et al., 1992; Rai et al., 2010). Consistently, dysregulated DNA methylation promotes tumorigenesis, neurodevelopmental disorders, and degenerative disease (Shames et al., 2007). DNA methylation interacts with Methyl-CpG binding protein 2 (MeCP2) (Fasolino and Zhou, 2017). MeCP2 consists of a methyl-CpG binding domain (MBD) and a transcription repression domain (TRD). Traditionally, MeCP2 regulates gene expression through its binding to methylated DNA in a sequence-specific manner via MBD and its interaction with corepressors through the TRD domain (Liyanage et al., 2019). DNA demethylation stimulates the release of MeCP2 from its target sequences and enhance gene expression. MeCP2 is expressed throughout the central nervous system (CNS) and mediates brain activity and development. MeCP2 silencing leads to the slower maturation of neurons and delayed synaptic formation, leading to Rett's syndrome (RTT) (Townend et al., 2018). In addition to its functionality in neuronal maturation, MeCP2 regulates NSCs/NPCs regeneration and differentiation (Tsujimura et al., 2009). Truncated versions of MeCP2 (R168X, lacking the TRD), in RTT patients, lead to a loss of MeCP2 functionality, highlighting its ability to regulate neural plasticity.

The posttranslational phosphorylation of MeCP2 is key to its cellular effects (Chao and Zoghbi, 2009; Tao et al., 2009). Phosphorylation of MeCP2 is the likely switch that controls transcription of specific target genes. Phosphorylation site-specific mutations will influence neighboring bases and secondary structures, promoting neurodysplasia and decreasing synapse numbers (Jugloff et al., 2005; Chao and Zoghbi, 2009; Tao et al., 2009). These changes occur in both RTT patients and MeCP2 mutant mice (Ananiev et al., 2011) and the neuro-phenotypic mutations may be related to MeCP2 phosphorylation defects. Therefore, it is essential to determine the functional significance of MeCP2 phosphorylation vis-à-vis its role in the brain and the effect of MeCP2 mutations on the neural outcomes of RTT.

The expression and phosphorylation of MeCP2 and the regulation of its activity have not been investigated or characterized in AD. Here, we defined these properties *in vivo* and assessed their roles in the differentiation direction of NSCs/NPCs. MeCP2 was observed to undergo dynamic *de novo* phosphorylation at serine 421 (S421) following AD injury, highlighting its functionality in early AD stages in the mouse brain. MeCP2 phosphorylation coupled to AD injury-induced DNA demethylation in *Gfap, Nestin*, and DCX promoters causing a loss of MeCP2 binding to its cellular targets, thereby relieving transcriptional repression, permitting gene activation. The epigenetic modifications in such lineage-specific gene promoters may be involved in the regeneration and fate determination of NSCs/NPCs in the striatum of AD mice following AD7c-NTP inhibition. This provides a platform to investigate the epigenetic mechanisms in regulating neurogenesis and differentiation in the AD mouse striatum.

## Experimental Procedures

### AD7c-NTP shRNA Interference

The complementary DNA (cDNA) plasmids encoding small-hairpin RNA (shRNA) interfering sequences were produced by Dharmacon Shanghai GenePharma. These shRNA plasmids product consists of a mixture of three to five lentiviral vector plasmids, each of which contains a target gene-specific coding for a 19–25 nt shRNAs, and a 6-bp folding region designed to stably inhibit AD7c-NTP gene expression. The other shRNA plasmid encodes a scrambled sequence that does not cause specific degradation of any known cellular messenger RNA (mRNA) sequence as a control for the off-target effects of shRNA (Dharmacon Shanghai GenePharma).

### Animal Protocols

#### Mouse Models

B6C3-Tg (APPswe, PSENlde9) 85Dbo/J transgenic mice (adult males) and matched wild-type mice were used in the study (Peking Experimental Animal Center of the Chinese Academy of Medical Sciences). All experiments were approved by our local ethics board in accordance with National Institute of Health Guide protocols approved by the Medical Experimental Animal Administrative Committee of Tianjin and with animal protocol approved by the Nankai University. To exclude the effect of sexual cycle on the results, only male mice were used for all experiments. Animal suffering was minimized, and the minimum number of animals were assessed. All animal experiments were conducted at the same time every morning.

#### Animal Surgery

Animals aged 3 months (to simulate the early stages of AD) with body weights of ~25 g were randomly divided into AD7c-NTP shRNA (NTP shRNA) and control shRNA (Ctr shRNA) groups. Animals were anesthetized with ketamine/xylazine, and arterial blood samples obtained via a femoral catheter were collected to measure the pO_2_, pCO_2_, and pH using the AVL 990 Blood Gas Analyzer (AVL List GmbH, Graz, Austria). The rectal temperature was maintained at 37 ± 0.5°C via a temperature-regulated heating lamp. Mice with physiological variables within normal ranges were subjected to the subsequent experimental studies. The plasmid preparation mixture containing 1 μg plasmids in 9 μl 0.9% sterile saline and 1 μl Lipofectamine 2000 [Cat no. 11668-109, Invitrogen (year 2018)] was stereotaxically delivered into the left ipsilateral ventricle (AP: −0.8 mm; ML: 1.4 mm; DV: −3.6 mm for dorsoventra). Mice were then returned to their cages following the recovery from anesthesia. Mice were provided free access to water and food. All animals were sacrificed after 2 weeks.

### Tissue Assessments

Mice were anesthetized to minimize animal suffering during the procedure. For fixation, saline and 4% paraformaldehyde (PFA) solution were intracardially perfused in 0.1 M phosphate-buffered solution (PBS, pH 7.4). Brains were extracted and fixed in 4% PFA for 6 h and immersed in a solution of 20–30% sucrose until sinking was observed. Coronal samples were sectioned (freezing microtome, Model 820-II) at 30 μm Bregma from 1.60 to −4.80 mm. Sections were cryostored at −20°C prior to histological analysis.

### Immunohistochemical Staining

For single staining, tissue sections were boiled for 13 min in trisodium citrate buffer at 97.9°C for antigen retrieval and probed with anti-MeCP2 (non-phosphorylated form, rabbit, 1:100; Cat no. 07-013; RRID: AB_2144004, Merck Millipore), antiphosphorylated MeCP2-S421 (MeCP2 pS421, rabbit, 1:100; Cat no. PA5-35396; RRID: AB_2552706, Abcam), or anti-AD7c-NTP [mouse, 1:100; Cat no. orb389662, Bioreagents, (year 2018)] antibodies. Sections were labeled with the appropriate biotinylated antibodies (1:200, Vector Laboratories) and incubated with avidin–biotin–peroxidase (1:200, Vectastain Elite ABC kit) for 1 h at 37°C. Signals were developed following diaminobenzidine (DAB, 0.5%) labeling. Primary antibody labeling was omitted in the negative control samples. To verify the specificity of MeCP2 pS421 antibody, the sections were probed with a mixture of phospho-MeCP2-S421 blocking peptide [at 10 times higher concentration than the antibodies, Cat no. BP3693a, ABGENT (year 2019)] and the anti-MeCP2 pS421 antibody preincubated at room temperature for 1 h. The rest of the dyeing procedure is the same as above.

For double staining of AD7c-NTP with 4′6-diamidino-2-phenylindole dihydrochloride (DAPI), the cerebral slices were sequentially incubated with the anti-AD7c-NTP antibody, biotinylated antimouse immunoglobulin G (IgG) (1:200), and avidin–biotin–peroxidase (1:200). The sections were then stained with Vector® Blue color solution for 30 min, rinsed with PBS, and further stained with DAPI (1 μg/ml; Invitrogen) for 10 min at room temperature. Following dehydration, clearing, and sealing, the sections were observed under a microscope.

### Fluorescence Immunolabeling and Confocal Microscopy

For dual immunostaining, sections were labeled with rabbit polyclonal anti-MeCP2 (1:100) or anti-MeCP2 pS421 (1:100) with goat polyclonal antiglial fibrillary acidic protein (anti-GFAP) (1:200; Cat no. ab53554; RRID: AB_880202, Abcam), mouse monoclonal anti-NeuN (1:500; Millipore, Cat. no. MAB377; RRID: AB_229877), mouse monoclonal anti-Nestin (1:500; Cat no. 556309; RRID: AB_396354, BD Biosciences), or goat polyclonal anti-DCX (1:100; Cat no. sc-8066; RRID: AB_2088, Santa Cruz). Sections were then labeled with antigoat IgG-rhodamine (1:40; Santa Cruz) or antimouse IgG–fluorescein isothiocyanate (FITC) (1:40; Santa Cruz) for 1 h at 37°C and mounted using fluorescent mounting media (Vector Laboratories).

The fluorescent double staining was performed by MeCP2, MeCP2 pS421, or AD7c-NTP with DAPI to explore their intracellular distribution. The sections were first incubated with the primary antibody against MeCP2, MeCP2 pS421, or AD7c-NTP and then with the secondary antibody as described above. After further stained with DAPI (1 μg/ml) for 10 min at room temperature, the sections were finally washed, mounted, and coverslipped.

All the sections were detected on a confocal microscope (TCS SP5, Leica, Heidelberg) at excitation of 650 nm and an emission of 670 nm (Cy5), 535 and 565 nm (rhodamine), 490 and 525 nm (FITC), and 358 and 461 nm (DAPI).

### Nuclear and Cytoplasmic Protein Extraction

Nuclear and cytoplasmic extracts were obtained using commercial kits (Beyotime Biotechnology). Fresh striatal tissue was dissected, homogenized, and treated with cytoplasmic protein extraction agent containing 1 mM phenylmethanesulfonyl fluoride (PMSF) for 15 min on ice. Samples were centrifuged at 1,500 *g* for 10 min at 4°C to separate the cytoplasmic components into the supernatants and the nuclei into the pellet fraction. Pellets were resuspended in protein extraction supplemented with PMSF, chilled on ice, and vortexed every 15 s every 10 min on three occasions. Samples were centrifuged (10 min at 16,000 *g*) and stored prior to assessments.

### Whole-Cell Protein Extraction

The brain tissue samples were dissected and homogenized in protein lysate and protease inhibitor PMSF buffer. After 30 min in an ice bath, the supernatant was collected after centrifuging at 13,000 *g* for 30 min at 4°C; then, the protein concentration was determined by bicinchoninic acid (BCA) method. All the samples were stored at −70°C for following Western blot analysis.

### Western Blotting

Samples were boiled for 5 min, resolved on 8% sodium dodecyl sulfate–polyacrylamide gel electrophoresis (SDS-PAGE) gels and transferred to polyvinylidene difluoride (PVDF) membranes (Bio-Rad). The membranes were blocked in 10% skimmed milk in Tris-buffered saline Tween (TBST) and probed overnight with anti-MeCP2 (1:1,000), anti-MeCP2 pS421 (1:1,000), anti-GFAP (1:1,000), anti-Nestin (1:1,000), anti-DCX (1:1,000), and anti-AD7c-NTP antibodies (1:1,000) at 4°C. Membranes were then washed and labeled with horseradish peroxidase (HRP)-conjugated secondary antibodies (1:3,000, Santa Cruz) for 1 h at room temperature, and bands were visualized using enhanced chemiluminescence (ECL). Bands were then quantitated and normalized to β-actin (1:10,000; Cat no. A5441; RRID: AB_476744, Sigma) as a loading control following stripping and reprobing. β-Tubulin (1:5,000; Cat no. T5293; RRID: AB_477580, Sigma) and histone 3 (H3) (1:1,000; Cat no. 9715; RRID: AB_331563, Cell Signaling Technology) were used as cytoplasmic and nuclear markers, respectively. Immunostained bands were quantified on Image J. Negative controls received identical treatment except for the absence of primary antibodies and showed no specific staining. Phospho-MeCP2-S421 blocking peptide was used as described in *Immunohistochemical Staining*.

Alkaline phosphatase (PPTase; New England Biolabs) was used to verify the specificity and effectiveness of the phosphorylated antibody. PPTase was added to the mixture of protein lysates and NE buffer (New England Biolabs) and incubated at 37°C for 30 min. After precipitating overnight in cold acetone at −20°C and centrifuging, the purified proteins were resuspended and used for subsequent immunoblotting analysis.

### Chromatin Immunoprecipitation Assays

For chromatin immunoprecipitation (ChIP) assessments, the striatum was dissected from adult rats and fixed in 1% formaldehyde to permit the cross-linking of DNA to proteins. Following glycine treatment, tissues were washed in ice-cold PBS (3×) supplemented with protease inhibitors (Roche) and homogenized. Following centrifugation, pellets were resuspended in shearing buffer plus protease inhibitors on an ultrasonic liquid processor (Qsonica) to generate chromatin fragments (average length: 400–800 bps on agarose gels). Chromatin was subjected to immunoprecipitation in strip wells and labeled with anti-MeCP2, anti-MeCP2 pS421, or an equivalent volumes of IgG. A portion of the samples was collected as input samples. After six washes in IP wash buffer, precipitated protein–DNA complexes were liberated through proteinase K treatment (15 min at 65°C) and incubated in reversing solution at 65°C for 90 min to dissociate the formaldehyde cross-links. Following cross-link reversal and DNA purification, quantitative polymerase chain reactions (qPCRs) were performed. The following primers were assessed: GFAP—for, 5′-TCAATAAAGGCCCTGACATCC-3′ and rev, 5′-AGCAGCCAAGAGGCTCTCCT-3′; Nestin—for, 5′-TCTTCTCTTCTGCACCCGGATG-3′ and rev, 5′-TCTTCCCCGACGCAACCCT-3′; DCX—for, 5′-CCCCTTACCCTTCCTTATTC-3′ and rev, 5′-CGGTCAGAAGAAACAGCGTACA-3′ (*n* = 6).

### Methylated DNA Immunoprecipitation Assays

Genomic DNA was extracted from mice striatum following overnight proteinase K treatment at 55°C, phenol–chloroform extraction, ethanol precipitation, and RNaseA treatment as previously described (Weber et al., 2005). Random genomic DNA fragments were produced via sonication (400–800 bp in length), and a total of 4 μg of fragmented DNA was assessed in methylated DNA immunoprecipitation (MeDIP) assays. DNA was denatured for 10 min at 95°C and immunoprecipitated for 2 h at 4°C with 10 μl of anti-5-methylcytidine antibodies at a final volume of 500 μl IP buffer [10 mM sodium phosphate (pH 7.0), 140 mM NaCl, 0.05% Triton X-100]. A sample of sonicated DNA was left untreated and served as an input control. Samples were incubated in 30 μl of protein A-Sepharose beads (GE healthcare) for 2 h at 4°C and washed 3× in IP buffer. Beads were collected and treated with proteinase K for 3 h at 50°C. Methylated DNA was extracted through phenol–chloroform extraction and ethanol precipitation, and samples were subjected to qPCR amplifications.

### Data Quantification and Statistical Analysis

Cell numbers were counted from four random confocal fields (thickness, 28 μm) randomly obtained per section in the striatum at a magnification of 40× object. Single- or double-labeled cells were counted manually, averaged and expressed as cells/mm^3^ (Chahrour et al., 2008, Turunen et al., 2009). Ct values were calculated for each PCR reaction and normalized to input IgG and quantified [2 – (ΔCt) antibody/2 – (ΔCt) IgG]. ΔCt values were calculated for each sample by subtracting the Ct value from the input (Ct Input) or from the Ct value of the immunoprecipitated sample (Ct antibody or Ct IgG). ΔCt antibody assessments: ΔCt IgG = [Ct antibody (or IgG) – Ct Input]. Data were plotted as relative enrichment over control groups.

The experiments were carried out by blind method, and the group assignment, operation, and analysis of experimental animals were carried out by a different person. Data are the mean ± SEM. Multiple genes were compared through a one-way ANOVA followed by least significant difference (LSD) assessment. Experimental groups were compared using an unpaired Student's *t*-test. *P* < 0.05 was considered statistically significant.

## Results

### AD Damage Induces MeCP2 Phosphorylation at S421 and Translocation From the Nucleus to Cytoplasm in Neural Cells

**Figure 1:**
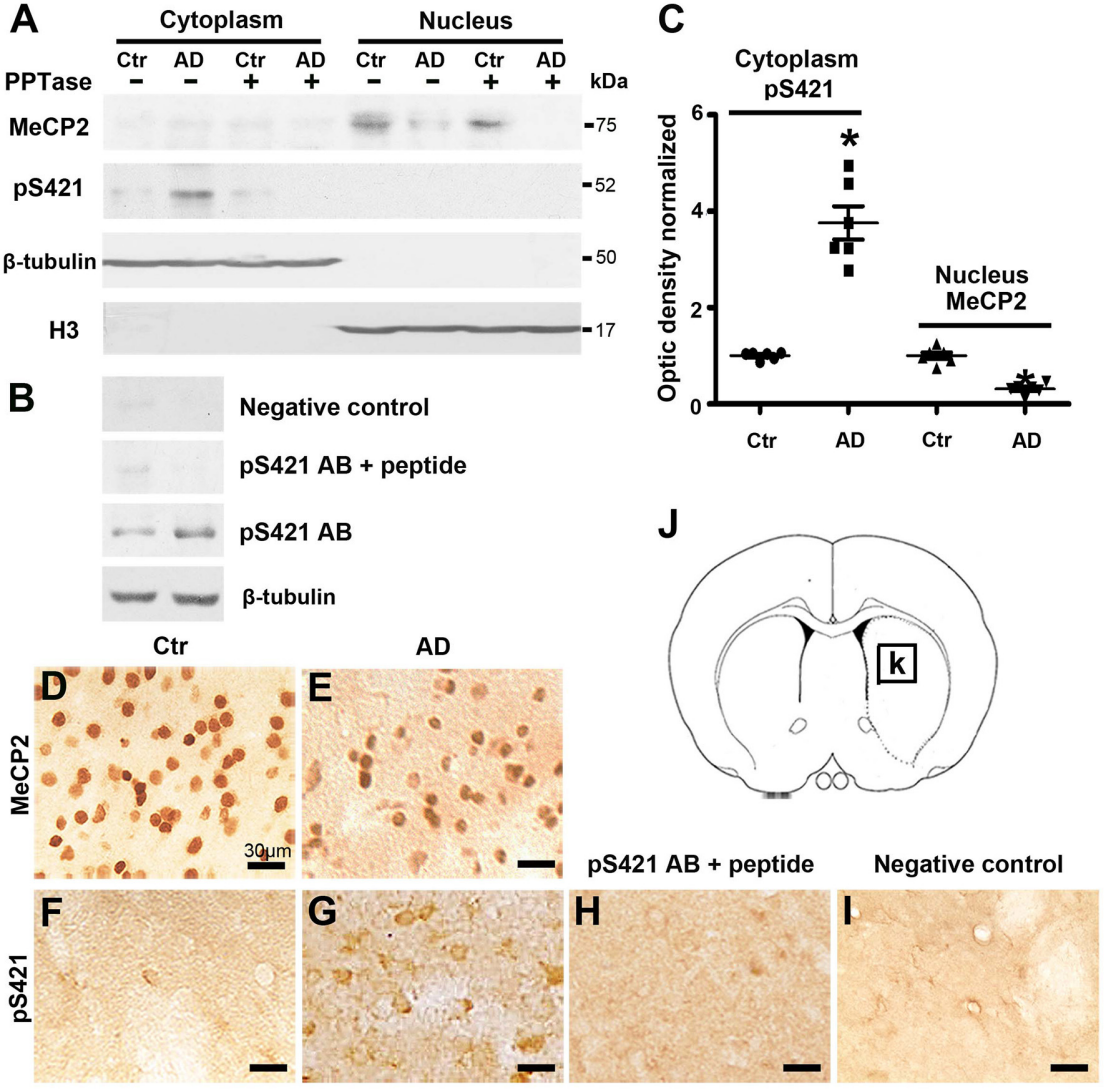
Changes in the expression of MeCP2 and MeCP2 pS421 in the adult mice striatum. **(A,B)** Alzheimer's disease (AD) mice striatum was collected at age of 3 months. Cytoplasmic proteins and nuclear proteins were extracted, respectively. Western blots were used to reveal MeCP2 and MeCP2 pS421 immunoreactivity and its normal controls. The data showed that MeCP2 selectively focused in the nucleus while MeCP2 pS421 in the cytoplasm. β-Tubulin and H3 were used for evaluating the loading control and the extracted purity of cytoplasmic and nuclear components severally. The specificity of the antibody to MeCP2 pS421 was validated by using both **(A)** PPTase and **(B)** phospho-MeCP2-S421 antibody-specific blocking peptide, and the immune-positive signals of MeCP2 pS421 was eliminated. The same PVDF membrane transfected with protein was used in this test. **(C)** Quantitative analysis revealed the decrease in MeCP2 expression levels in the nucleus and increase in MeCP2 pS421 expression levels in the cytoplasm in AD mice striatum. **(D–G)** Single immunostaining of MeCP2 and MeCP2 pS421 in the mice striatum. **(D)** MeCP2 was widely distributed in normal striatal cells. **(E)** AD injury reduced its immunoreactivity in the same viewing areas. While MeCP2 pS421 was detected highly in **(G)** AD mice striatal cells but barely in the **(F)** controls. **(H)** The blocking peptide eliminates the anti-phospho-MeCP2-S421 signals. **(I)** Negative control without primary antibody. **(J)** Diagram illustrates the region for observation [rectangle, **(K)**] (mean ± SEM, *P < 0.05 vs. control group mice, *n* = 6 in each group).

MeCP2 is exclusively localized to the nucleus but translocates to the cytoplasm when phosphorylated (Miyake and Nagai, 2007). To assess the intracellular distribution and expression levels of MeCP2 and MeCP2 pS421 after transgenic modification, we isolated nuclear and cytoplasmic extracts from the striatum tissue and performed Western blot analysis. MeCP2 was expressed exclusively in the nuclear fraction but not in the cytoplasmic fraction (~75 kDa), whereas MeCP2 pS421 was detected exclusively in the cytoplasmic fraction (Figure 1A). In addition, a loss of MeCP2 in the nuclear fraction and elevated levels of MeCP2 pS421 in the cytoplasmic fraction were observed in APP/PSl transgenic mice (Figure 1C). Furthermore, both PPTase and phospho-MeCP2 S421 antibody blocking peptide obliterated the band for MeCP2 pS421, thus proving the effectiveness and specificity of the pS421-targeting antibody (Figures 1A,B).

Subsequently, immunohistochemical (IHC) was used to analyze the distribution of MeCP2 and MeCP2 pS421 in adult mice striatum. The immunoreactivity of MeCP2 and MeCP2^+^ cell numbers were both significantly reduced in the APP/PSl transgenic mice striatum (Figures 1D,E). Simultaneously, we observed an increased MeCP2 pS421^+^ expression in the APP/PSl transgenic mice striatum (Figure 1G), which were weakly detected in control groups (Figure 1F). In addition, preincubation with the blocking peptide neutralized MeCP2 pS421 staining (Figure 1H). Staining without primary antibody was used as negative control (Figure 1I). Diagram illustrates the region for observation (Figures 1J,K).

### AD Injury Alters the Expression Patterns of MeCP2 and MeCP2 pS421 in Adult Mice Striatum

**Figure 2:**
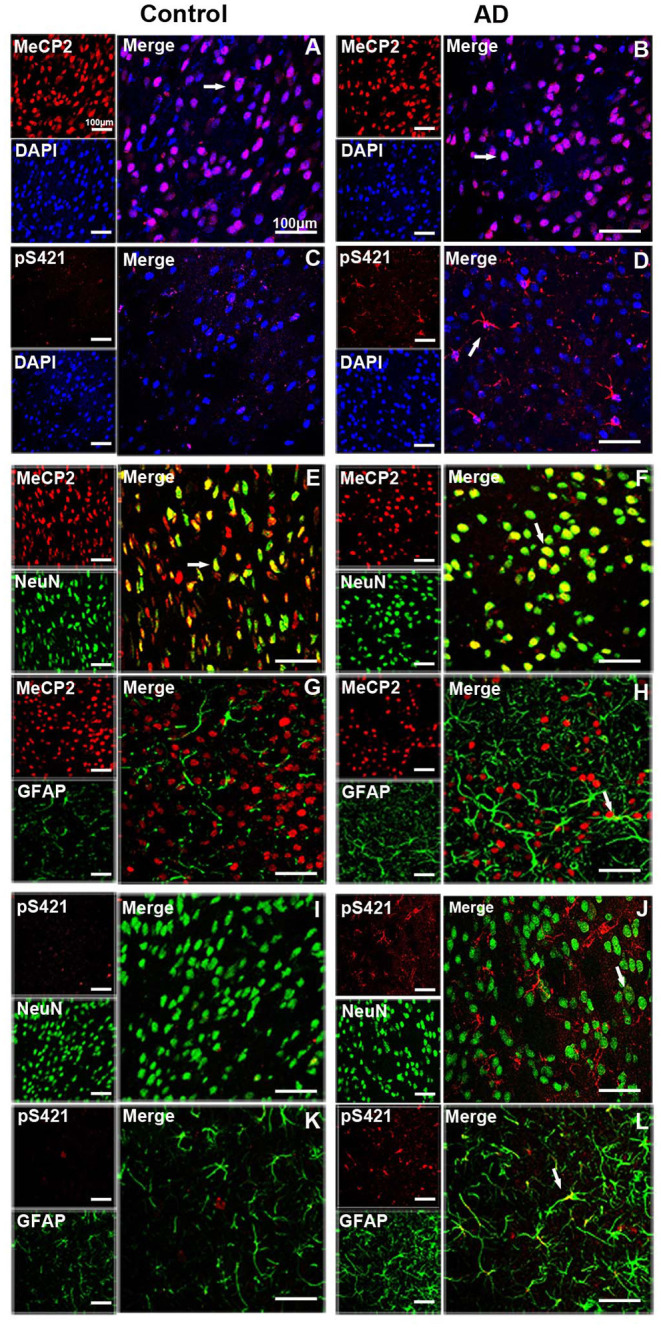
Expression patterns of MeCP2 and MeCP2 pS421 in the adult mice striatum. **(A–D)** Intracellular localization of MeCP2 and MeCP2 pS421 in mice striatum. **(A,D)** Immunofluorescent double staining of MeCP2 or MeCP2 pS421 with DAPI. MeCP2 was colocalized with DAPI in the nucleus of both normal mice and Alzheimer's disease (AD) mice. **(C,D)** MeCP2 pS421 was mainly distributed in the nucleus. **(E–L)** Cellular types expressing MeCP2 and MeCP2 pS421 in mice striatum. Immunofluorescent double staining showed that MeCP2 was strongly expressed **(E)** in NeuN^+^ neurons, but barely in **(G)** GFAP^+^ astroglia in the normal striatum. However, MeCP2-positive signals not only existed in **(F)** NeuN^+^ neurons but also in **(H)** GFAP^+^ astroglia in the AD mice striatum. Immunofluorescent double staining revealed elevated MeCP2 pS421-positive signals colocalized with **(L)** GFAP^+^ signals and with **(J)** NeuN^+^ signals to a lesser extent in the AD mice striatum. MeCP2 pS421-positive signals in the normal striatum were much weaker **(I,K)**. The arrows indicate a double positive cell.

MeCP2 was mainly colocated with DAPI and concentrated in the nucleus in both normal and AD brain (Figures 2A,B), while MeCP2 pS421was largely cytoplasmic in AD brain (Figure 2D), which was consistent with the results of Western blotting (Figure 1A). Furthermore, MeCP2 was concentrated in the NeuN^+^ neurons of the normal striatum (Figure 2E) but barely detected in the GFAP^+^ astroglia (Figure 2G). In the AD striatum, however, MeCP2 was present in both neurons (Figure 2F) and GFAP^+^ astroglia (Figure 2H). It is worth noting that AD-induced damage elevated MeCP2 pS421 protein was chiefly localized in GFAP^+^ astroglia (Figure 2L), and some protein signals were detected in NeuN^+^ neurons as well (Figure 2J). The expression level of MeCP2 pS421 in normal mice brain can barely be detected (Figures 2C,I,K). These results suggest that AD alters the intracellular localization and cell-type-specific distribution of MeCP2 and MeCP2 pS421.

### AD7c-NTP Silencing Induces MeCP2 S421 Phosphorylation in APP/PSl Transgenic Mice Striatum

**Figure 3:**
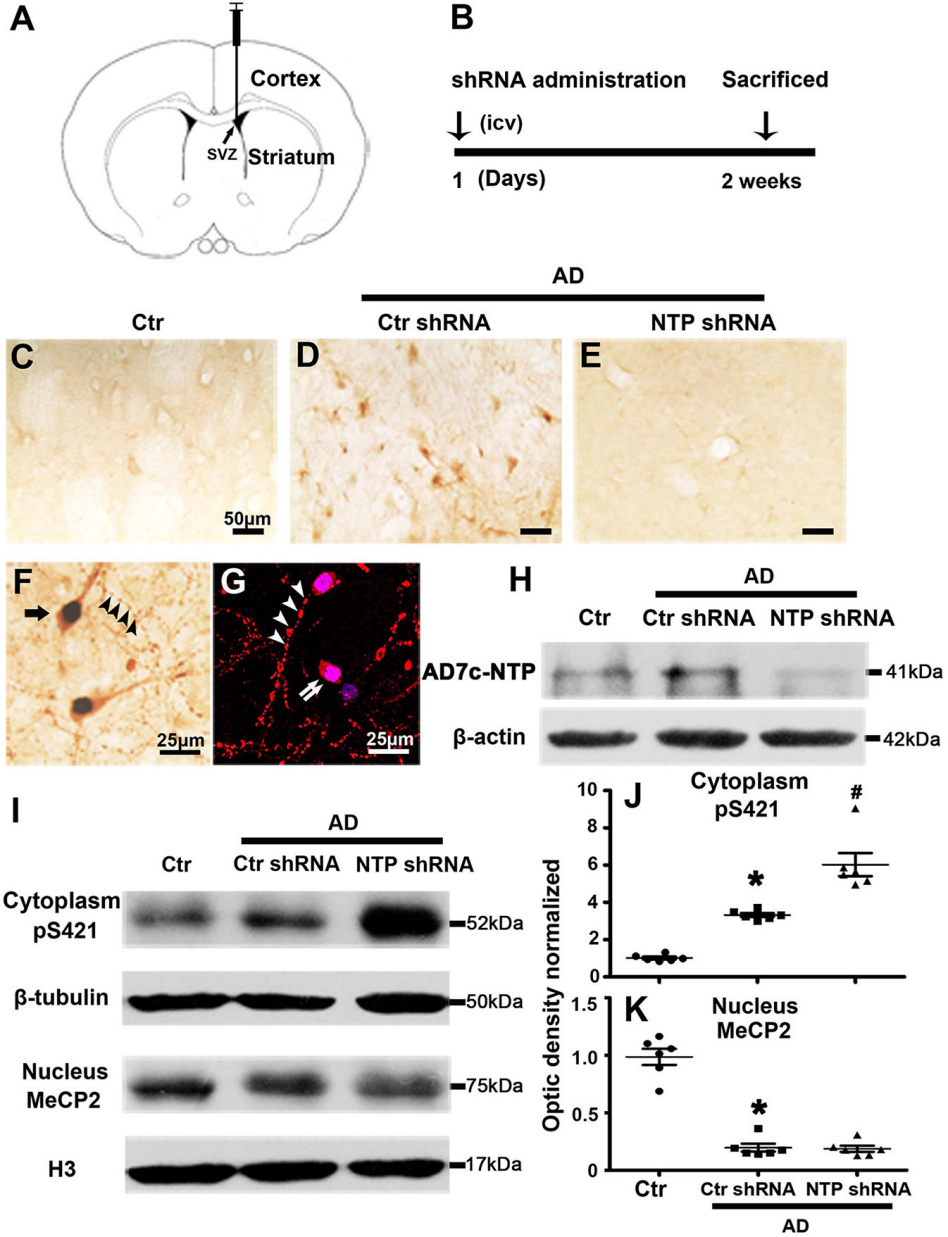
MeCP2 pS421 is upregulated in Alzheimer's disease (AD) mice striatum after AD7c-NTP small-hairpin RNA (shRNA) treatment. Mice striatum was collected at age of 3 months. Cytoplasmic proteins and nuclear proteins were extracted, and protein levels in each fraction were assessed. **(A,B)** The timeline diagram of this study. Animals received AD7c-NTP shRNA or scrambled shRNA injection into lateral ventricle at age of 3 months and were sacrificed 2 weeks later. **(C–E)** Altered AD7c-NTP immunoreactivity in AD mice. Immunohistochemical staining showed enhanced expression of AD7c-NTP in **(D)** AD mice relative to **(C)** aged control and **(E)** inhibited by AD7c-NTP shRNA. **(F,G)** Immunohistochemical double labeling [**(F)**, brown: AD7c-NTP; blue: 4′6-diamidino-2-phenylindole dihydrochloride (DAPI)] and immunofluorescence double labeling [**(G)**, red: AD7c-NTP; blue: DAPI)] showed increased AD7c-NTP in AD striatal neurons (single arrow), as well as in degenerating neurons (double arrows) and abnormal neuritic processes (arrowheads). **(H)** Western blot analysis showing downregulation of endogenous AD7c-NTP in AD mice. **(I–K)** Western blot analysis showed that AD7c-NTP shRNA injection has further enhanced the cytoplasmic protein levels of MeCP2 pS421 compared with the scrambled plasmid treatment groups **(I)**. **(I)** While AD7c-NTP shRNA injection has no impact on the MeCP2 protein levels in nucleus. Quantitative analysis of **(J)** MeCP2 and **(K)** MeCP2 pS421 protein levels, respectively (mean ± SEM, *P < 0.05 vs. control group mice, ^#^*P* < 0.05 vs. AD + Ctr shRNA group mice, *n* = 6 in each group).

We observed altered MeCP2 and MeCP2 pS421 expression following AD injury. We further investigated the effects of AD7c-NTP on the expression of MeCP2 and MeCP2 pS421 by injecting AD7c-NTP shRNA and scrambled shRNA plasmids into the lateral ventricle of APP/PSl transgenic mouse brains. All the animals were sacrificed 2 weeks later (Figures 3A,B). First, the data of immunohistochemistry and Western blot both demonstrated that the expression level of AD7c-NTP in the AD7c-NTP shRNA group was significantly inhibited compared to the scrambled control group (Figures 3C–E,H), indicating successful inhibition of the endogenous AD7c-NTP. Furthermore, AD7c-NTP immunoreactivity was localized to the neurons, neuropil fibers, and irregular neuritic processes in the AD brains (Figures 3F,G). Western blotting also showed that AD7c-NTP shRNA treatment further increased the levels of cytoplasmic MeCP2 pS421 in the striatum compared to scrambled shRNA alone treatment groups (Figures 3I,J). However, AD7c-NTP shRNA treatment failed to alter the expression of nuclear MeCP2 (Figures 3I,K).

### AD7c-NTP Silencing Increases GFAP, Nestin, and DCX Expression in the Striatum of APP/PSl Transgenic Mice

**Figure 4:**
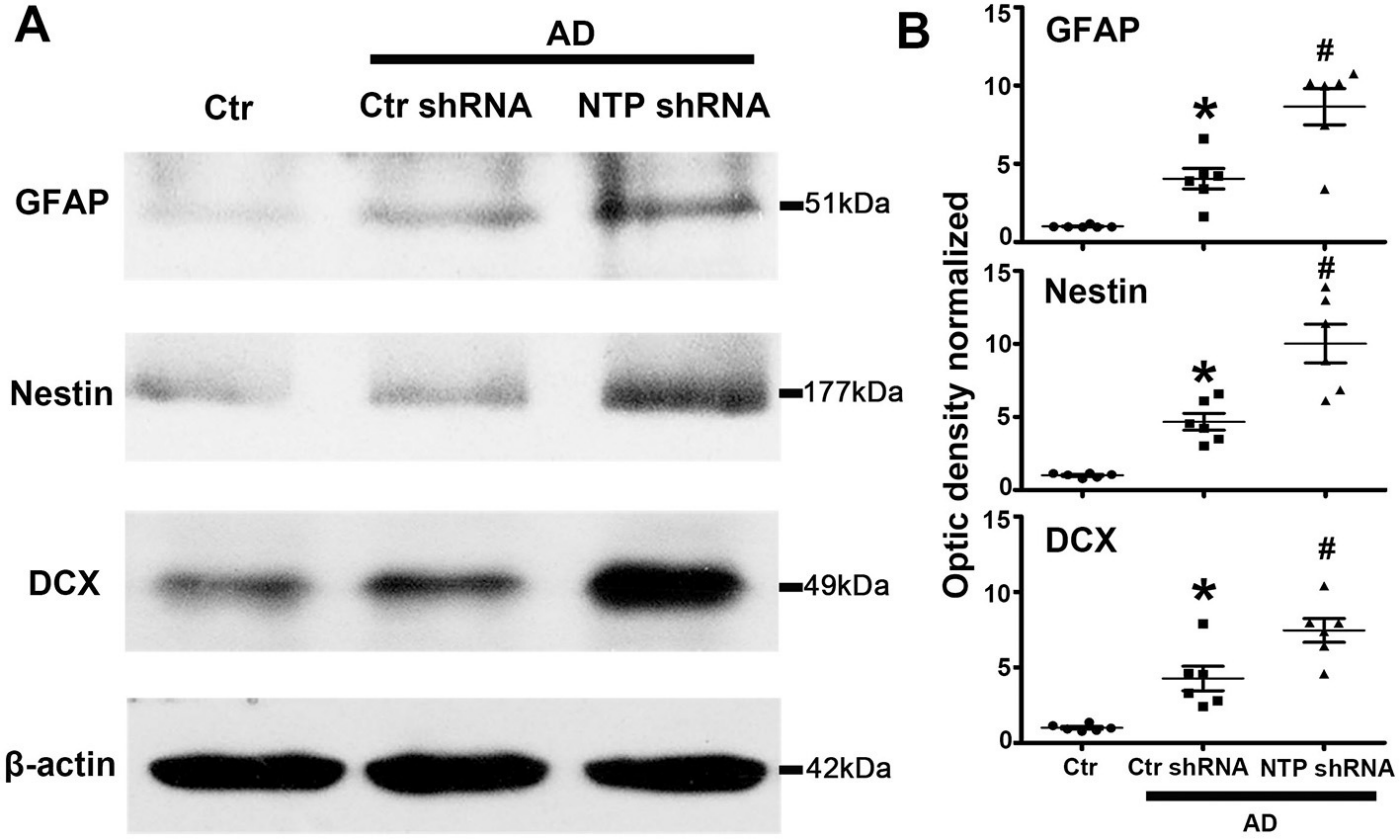
Further increasing of glial fibrillary acidic protein (GFAP), Nestin, and doublecortin (DCX) protein levels in Alzheimer's disease (AD) mice striatum after AD7c-NTP small-hairpin RNA (shRNA) treatment. **(A)** Western blots were employed to evaluate the protein expression of GFAP, Nestin, and DCX in AD mice at age of 3 months. The data showed that AD7c-NTP shRNA injection has dramatically enhanced GFAP, Nestin, and DCX protein levels compared with the scrambled plasmid treatment groups. **(B)** Quantitative analysis of these protein levels, respectively (mean ± SEM, *P < 0.05 vs. control group mice, ^#^*P* < 0.05 vs. AD + Ctr shRNA mice, *n* = 6 in each group).

The expression of GFAP, Nestin, and DCX was significantly lower in the normal control mice striatum. By contrast, the expression of all the proteins was remarkably elevated following AD damage. Moreover, AD7c-NTP shRNA treatment further increased GFAP, Nestin, and DCX expression compared to control mice (Figures 4A,B).

### DNA Demethylation and MeCP2 Phosphorylation at S421 Regulate the Activation of *Gfap, Nestin*, and *Dcx* Gene Expression

**Figure 5:**
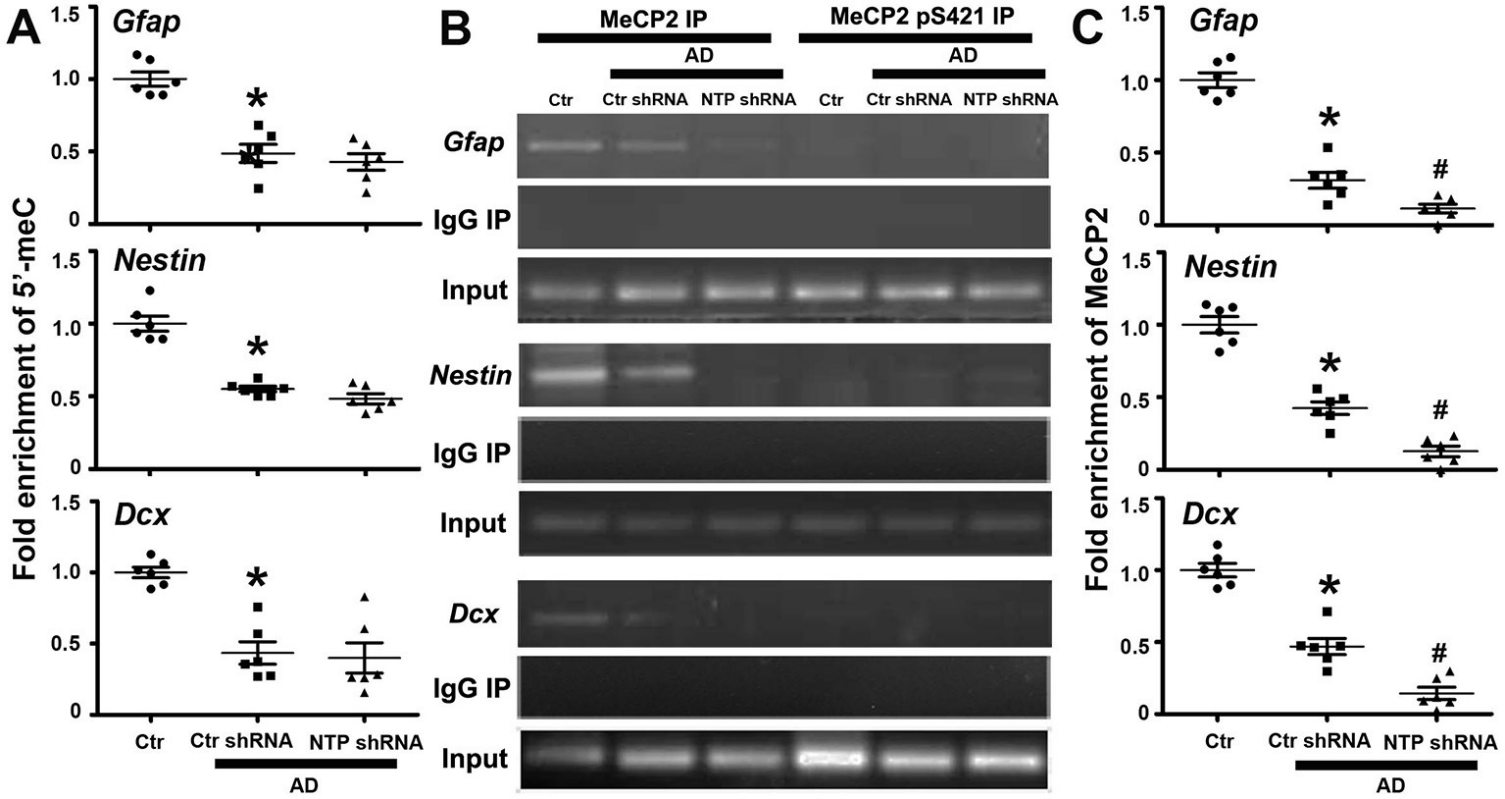
Epigenetic analysis of *Gfap, Nestin*, and *Dcx* gene promoter regions following AD7c-NTP small-hairpin RNA (shRNA) treatment in Alzheimer's disease (AD) mice striatum. **(A)** Methylated DNA immunoprecipitation (MeDIP) analysis using antibody specific to 5′-meC coupled with quantitative PCRs (qPCRs) amplification to assess the methylation status of *Gfap, Nestin*, and *Dcx* gene promoters. In control striatal cells, the gene promoters were extensively methylated. AD damage has dramatically decreased the methylation degrees of the same investigated regions. However, AD7c-NTP shRNA treatment did not affect the methylation levels of these sequences striking compared with the scrambled plasmid treatment groups. **(B)** Chromatin immunoprecipitation (ChIP) analysis using antibodies specific to MeCP2 or MeCP2 pS421 to assess the relative binding of MeCP2 or MeCP2 pS421 to the promoter regions of mice *Gfap, Nestin*, and *Dcx* genes. MeCP2 bound to these promoters tightly in the control striatal cells, whereas MeCP2 pS421 did not bind to the same investigated gene sequences. Normal mouse immunoglobulin G (IgG) was used as negative control, while MeCP2 immunoprecipitation was used for positive control. **(C)** ChIP-qPCRs analysis showed that AD injury attenuated the bonding force between MeCP2 and *Gfap, Nestin*, and *Dcx* gene promoters. AD7c-NTP shRNA treatment further weakened their association affinity tremendously. qPCRs values were normalized to the input and plotted as relative enrichment over control groups (mean ± SEM, *P < 0.05 vs. control group mice, ^#^*P* < 0.05 vs. AD + Ctr shRNA group mice, *n* = 6 in each group).

As epigenetic modifications regulate cell-type-specific gene expression, we reasoned that this may be responsible for the gene-specific regulation of *Gfap, Nestin*, and *Dcx*. We therefore examined their DNA methylation status via MeDIP assays with antibody specific for 5′-meC and determined whether the regional changes in DNA methylation correlated with the regulation of gene expression. We found that the regions around the transcriptional start sites were extensively methylated in normal control striatal cells, while AD injury dramatically decreased the levels of methylation of the same investigated regions. However, AD7c-NTP shRNA treatment produced no effects on the DNA methylation status of *Gfap, Nestin*, and *Dcx* promoters in the AD striatal cells (Figure 5A).

Epigenetic MeCP2 regulates gene expression by binding to methylated CpG dinucleotides via its MBD (Fasolino and Zhou, 2017). The regions around *Gfap, Nestin*, and *Dcx* transcription initiation sites were extensively methylated, inferring them as binding sites for MeCP2. We next investigated whether these promoter regions are modulated by MeCP2 using the ChIP method. As anticipated, anti-MeCP2, but not antimouse IgG, could pull down the gene sequences in control striatal cells, indicating that MeCP2 strongly bound to *Gfap, Nestin*, and *Dcx* promoters under resting conditions. We further found that MeCP2 pS421 failed to bind to the same detected promoter sequences of these genes (Figure 5B). Moreover, the combination of MeCP2 to *Gfap, Nestin*, and *Dcx* promoter regions was significantly attenuated following AD damage. AD7c-NTP shRNA treatment led to a further reduction in MeCP2 binding to these promoter regions (Figures 5B,C).

### Inhibiting AD7c-NTP Expression Reduces the Distribution of MeCP2 in Neural Cells

**Figure 6:**
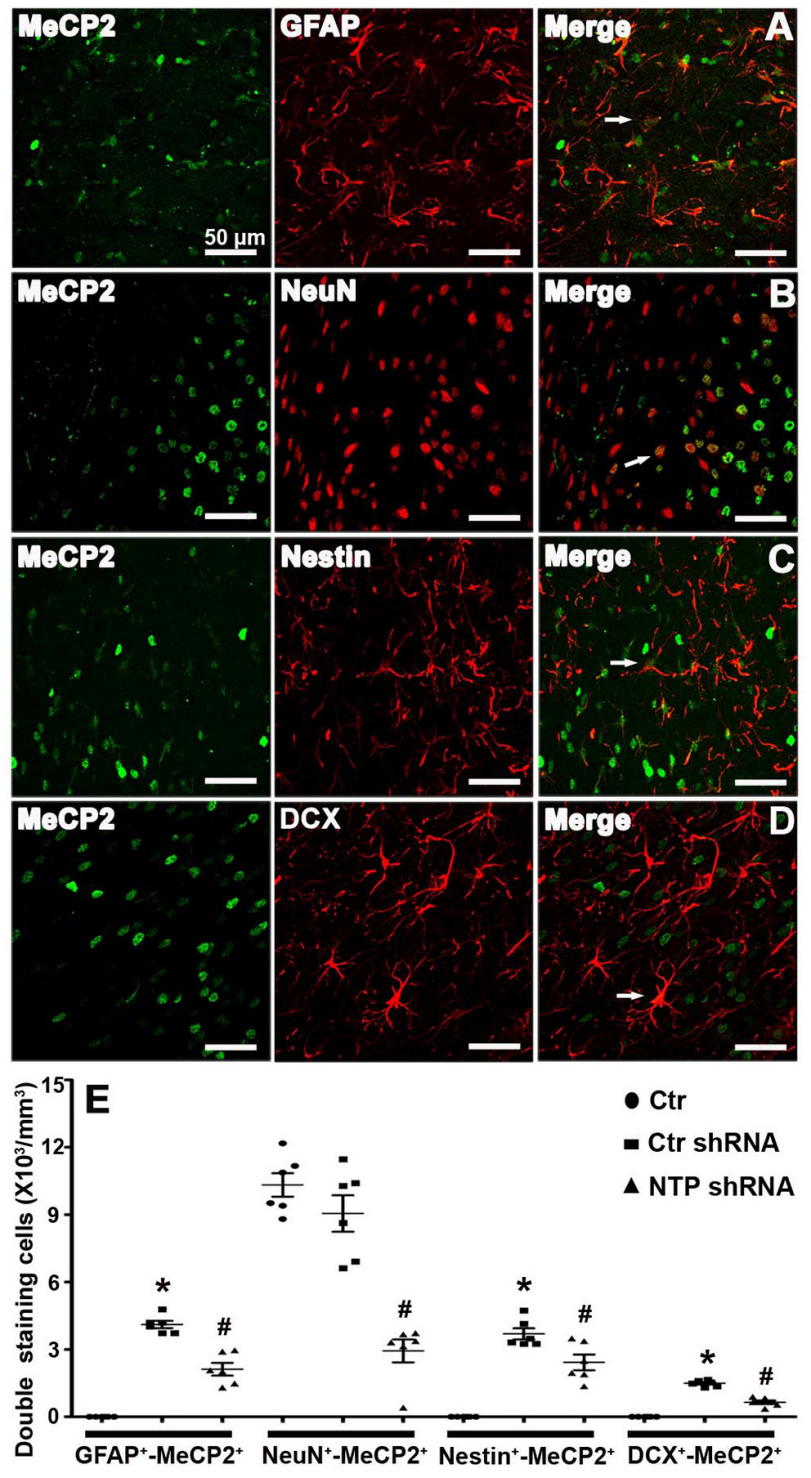
Colocalization of MeCP2 with glial fibrillary acidic protein (GFAP), NeuN, Nestin, and doublecortin (DCX) in the mice striatum at age of 3 months. **(A–D)** Confocal microscopy showed that MeCP2 signals can colocalize with GFAP^+^, NeuN^+^, Nestin^+^, and DCX^+^ cells in the striatum areas of Alzheimer's disease (AD) mice after AD7c-NTP small-hairpin RNA (shRNA) treatment. **(E)** Cell counting and statistical results of double-staining cells reveals barely GFAP^+^-MeCP2^+^, Nestin^+^-MeCP2^+^, and DCX^+^-MeCP2^+^ cells but a large number of NeuN^+^-MeCP2^+^ cells in the striatum of control mice. The number of GFAP^+^-MeCP2^+^, Nestin^+^-MeCP2^+^, and DCX^+^-MeCP2^+^ cells were increased in AD mice; however, AD7c-NTP shRNA treatment remarkably decreased the double staining neural cell numbers in the same brain region (mean ± SEM, *P < 0.05 vs. control group mice, ^#^*P* < 0.05 vs. AD + Ctr shRNA group mice, *n* = 6 in each group). The arrows indicate a double positive cell.

To directly assess whether MeCP2 regulates the neurogenesis of astroglia and neuronal developmental stages, cells were double immunostained for MeCP2 with GFAP, NeuN, Nestin, or DCX in brain sections. MeCP2 was found to be strongly expressed in NeuN^+^ neurons but was barely expressed in GFAP^+^ astrocytes, Nestin^+^ neural stem cells, or DCX^+^ immature neurons in the normal striatum (Figure 6E). However, MeCP2 colocalized with GFAP^+^, Nestin^+^, and DCX^+^ (GFAP^+^-MeCP2^+^, Nestin^+^-MeCP2^+^, and DCX^+^-MeCP2^+^ cells) in the APP/PSl transgenic mice (Figures 6A–D). Cell counting analysis showed that the number of GFAP^+^-MeCP2^+^, Nestin^+^-MeCP2^+^, and DCX^+^-MeCP2^+^ cells increased significantly in the striatum of APP/PSl transgenic mice, while that of NeuN^+^-MeCP2^+^ remained almost unchanged (Figure 6E). In contrast, AD7c-NTP shRNA treatment dramatically reduced all these double-labeled positive cell numbers in the same observational regions (Figure 6E).

### AD7c-NTP Silencing Redistributes MeCP2 pS421 in Neural Cells

**Figure 7:**
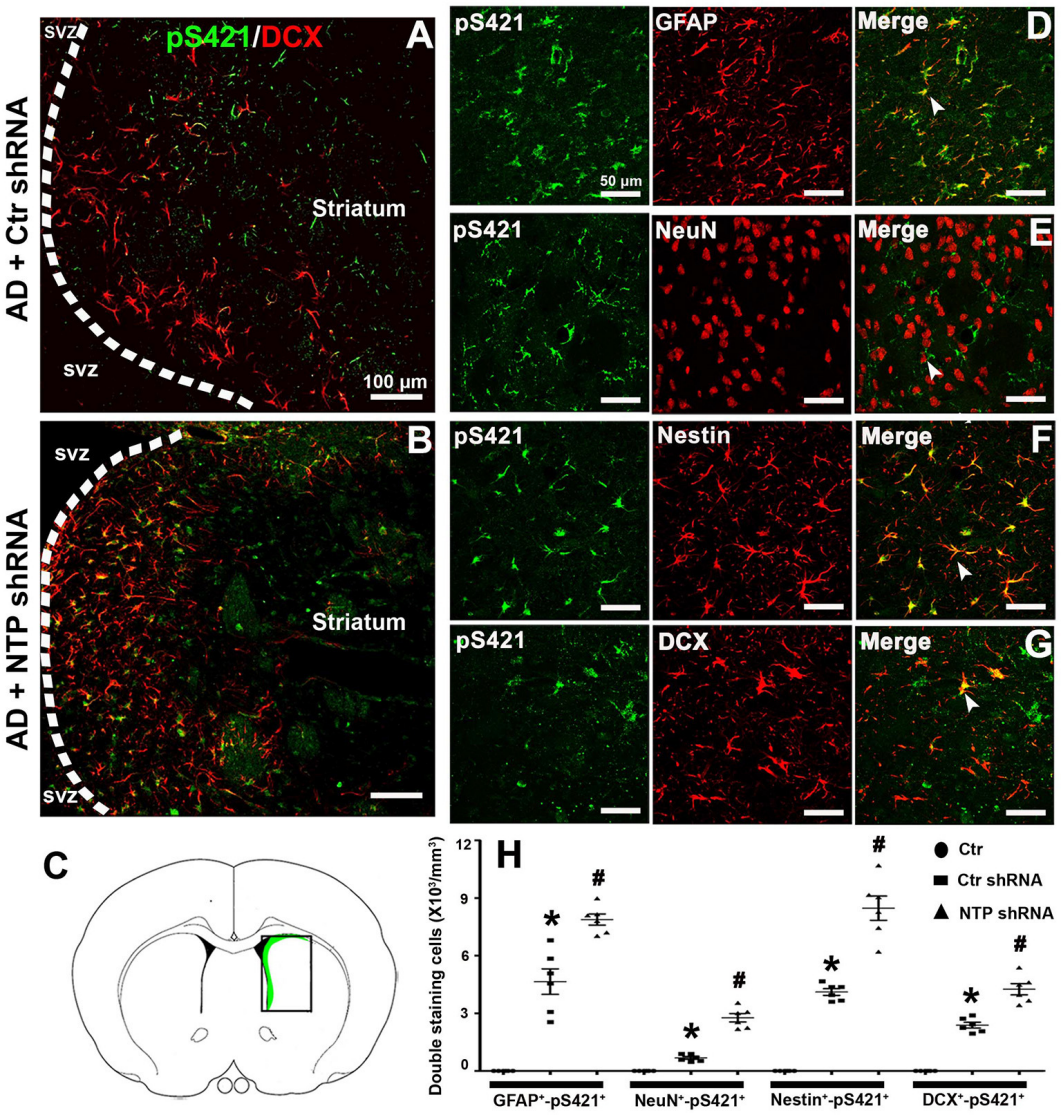
Colocalization of MeCP2 pS421 with glial fibrillary acidic protein (GFAP), NeuN, Nestin, and doublecortin (DCX) in the mice striatum at age of 3 months. **(A,B)** Microphotographs from the representative mice subjected to Alzheimer's disease (AD) damage showed a chain migration of DCX^+^-MeCP2 pS421^+^ cells that crossed the border between the subependymal ventricular zone (SVZ) and the adjacent striatum to the core striatum **(A)**. **(B)** AD7c-NTP small-hairpin RNA (shRNA) treatment was able to expand DCX^+^-MeCP2 pS421^+^ signal zone. **(C)** The rectangle in the diagram of the brain hemisphere indicates where images were acquired. **(D–G)** Immunofluorescence results showed overlapping expression of MeCP2 pS421 with GFAP, NeuN, Nestin, and DCX in AD mice striatum after AD7c-NTP shRNA treatment. **(H)** Cell counting and statistical results of double-staining cells (mean ± SEM, *P < 0.05 vs. control group mice, ^#^*P* < 0.05 vs. AD + Ctr shRNA group mice, *n* = 6 in each group). The arrows indicate a double positive cell.

Double staining revealed that MeCP2 pS421 markedly colocalized with GFAP^+^, Nestin^+^, and DCX^+^ (GFAP^+^-MeCP2 pS421^+^, Nestin^+^-MeCP2 pS421^+^, and DCX^+^-MeCP2 pS421^+^ cells) and to a lesser extent with NeuN^+^ (NeuN^+^-MeCP2 pS421^+^ cells) in the APP/PSl transgenic mouse model (Figure 7H). AD7c-NTP shRNA treatment significantly increased cell numbers compared to scrambled control groups (Figures 7D–G,H). In addition, DCX^+^-MeCP2 pS421^+^ cells formed chain-like structures extending from the SVZ to the striatum core. The DCX^+^-MeCP2 pS421^+^ cells localized to the striatum and exhibited a bipolar morphology with a long leading process or astroglia-like morphologies (Figures 7A,B). The rectangle in the diagram of the brain hemisphere indicates the region of interest (Figure 7C).

## Discussion

Neurogenesis requires integration of both intrinsic and extrinsic cues to establish intact circuits. The mechanisms of these processes are, however, not completely understood. MeCP2 binds to methylated DNA to regulate gene expression during neurogenesis and differentiation (Mehler, 2008). In this study, we demonstrate that AD7c-NTP is a regulatory factor for nerve regeneration in AD mice. We further show that AD damage triggers the *de novo* phosphorylation of MeCP2 at Serine 421 (S421) in the mouse striatum. MeCP2 phosphorylation in addition to DNA demethylation is essential to the regulation of *Gfap, Nestin*, and *Dcx* gene expression. Notably, we found that AD7c-NTP inhibition further induced the phosphorylation of MeCP2 at S421 and attenuated the affinity of MeCP2 for gene promoters, further enhancing gene expression. These epigenetic modifications in specific gene promoters represent an important signaling cue in the neurogenic reactions of neural cells following AD7c-NTP inhibition.

Adult neurogenesis requires further assessments in AD. Changes in the rates of cell division, differentiation, and viability have been reported in mice harboring APP and tau mutations (Chuang, 2010; Unger et al., 2016; Wirths, 2017). Cell proliferation is enhanced during differentiation in SGL and SVZ in AD-linked APP transgenic mice (Jin et al., 2004a,b; Lopez-Toledano and Shelanski, 2004, 2007; Herran et al., 2015). In humans, enhanced levels of proliferation and immature neuronal markers are observed (Nagy et al., 1997; Jin et al., 2004c; Boekhoorn et al., 2006). The data in this study inferred that NSCs/NPCs affect neurogenic areas of the brain in AD patients, contributing to hippocampal- and olfaction-dependent memory defects during the AD progression. The re-expression of neural stem cell marker protein Nestin indicates that the cells have regained their regenerative potential and have immature phenotype after AD injury (Cramer and Chopp, 2000). The downregulation of Nestin protein expression and upregulation of migrating neuronal precursor cell marker protein DCX following AD7c-NTP inhibition mean that the progenitor cells gradually break away from the proliferative state and differentiate into the mature state (Francis et al., 1999; Gleeson et al., 1999). De la Monte and colleagues showed the overexpression of AD7c-NTP in AD during early disease stages. Increased levels of AD7c-NTP in neurons enhance its association with phosphorylated tau protein (p-tau) (Monte et al., 1997). The overexpression of AD7c-NTP increases apoptotic cell death, mitochondrial dysfunction, and enhanced levels of CD95 and p53 (De la Monte and Wands, 2001b).

Some important questions still remain unsolved including the following; how does AD7c-NTP influence AD progression, and what pathways regulate the pathophysiology of AD? We propose an epigenetic mechanism including DNA methylation in concert with MeCP2 to describe the regulatory mechanisms of AD7c-NTP. MeCP2 promotes the regeneration of NSCs/NPCs by selectively or exclusively regulating cell-type-specific genes. However, R168X truncations of MeCP2, lacking the TRD, have been observed in RTT patients and are non-functional (Tsujimura et al., 2009; Fares et al., 2019). We found that MeCP2 undergoes dynamic *de novo* phosphorylation at a specific amino acid residue, Serine 421, in response to AD injury. Phosphorylated MeCP2 is present in the cytoplasm, while unphosphorylated MeCP2 localizes to the nucleus. We assessed the AD-induced loss of MeCP2 in the nucleus and the AD-induced increase in MeCP2 pS421 in the cytoplasm. This highlighted MeCP2 phosphorylation as a nuclear export signal that mediates the shuttling of MeCP2 from the nucleus to the cytoplasm following AD damage.

MeCP2 binds to methylated DNA to suppress gene expression and normal neural development (Rutlin and Nelson, 2011). The fact that AD-injury-induced MeCP2 phosphorylation at S421 alters its spatial distribution suggests that MeCP2 may function in a novel manner in the nervous system following AD injury. We considered how phosphorylation modulates the molecular function of MeCP2 during neurogenesis. One plausible model is that, in resting neural cells, MeCP2 acts as a transcriptional repressor of lineage-specific genes (including *Gfap, Nestin*, and *Dcx*) to repress their transcription. In the context of AD injury, DNA demethylation and MeCP2 phosphorylation at S421 reduce the binding of MeCP2 to these promoters, relieving transcriptional repression and permitting gene activation that controls the fate specification of NSCs/NPCs. AD7c-NTP shRNA silencing amplified MeCP2 pS421 expression in the cytoplasm and further attenuated the binding of MeCP2 to *Gfap, Nestin*, and *Dcx* promoters compared to control groups, thus further validated the model. This hypothesis is consistent with previous studies in which the phosphorylation of S421 released MeCP2 from the *Bdnf* promoter, facilitating *Bdnf* gene transcription, dendritic patterning, and spine morphogenesis (Zhou et al., 2006; KhorshidAhmad et al., 2016). However, recent studies show that MeCP2 is ubiquitously expressed throughout the mouse genome (Skene et al., 2010) and acts not only as a gene-specific but also as a global transcription factor (Skene et al., 2010; Cohen et al., 2011; Singleton et al., 2011). It has a bidirectional regulatory effect on target genes (Ben-Shachar et al., 2009; Urdinguio et al., 2010). MeCP2 phosphorylation regulates the genome-wide responses of chromatin during central nervous system development (Cohen et al., 2011). In addition, phosphorylation of MeCP2 may transiently alleviate the condensed chromatin structure and thereby facilitate proper gene expression (Skene et al., 2010). More reports show that site-specific phosphorylation of MeCP2 acts as a signaling transduction factor in neuronal gene expression and maintenance (Tao et al., 2009).

Our finding that MeCP2 phosphorylation at S421 activates specific genes expression explains the effects of MeCP2 on the regulation of NSCs/NPCs regeneration and suggests that MeCP2 phosphorylation is a crucial intrinsic cue for the neurogenic fate of NSCs/NPCs. This was supported by cell counting assays that demonstrated that 69.4 ± 2.3% DCX^+^ cells are MeCP2 pS421 positive, while 6.7 ± 1.8% DCX^+^ cells are MeCP2 positive after AD7c-NTP shRNA injection. Second, we demonstrated that AD7c-NTP silencing increases GFAP^+^, Nestin^+^, and DCX^+^ cell numbers but decreases GFAP^+^-MeCP2^+^, Nestin^+^-MeCP2^+^, and DCX^+^-MeCP2^+^ numbers in the same brain regions. This certified that the NSCs/NPCs acquired a neurogenic lineage with high MeCP2 pS421 expression (DCX^+^-MeCP2 pS421^+^, 79.0 ± 4.1%) but low total MeCP2 levels (DCX^+^-MeCP2^+^, 4.3 ± 1.1%) following AD7c-NTP silencing. The gain-of-MeCP2 phosphorylation function and loss-of-MeCP2 function showed prominent neurogenic effects in NSCs/NPCs in the AD mice striatum. The loss of MeCP2 pS421 in dormant NSCs/NPCs of the adult CNS may explain their neurogenic potential. However, it is possible that a range of uncharacterized modifications coordinate to regulate the function of MeCP2 during the regeneration and fate specification of NSCs/NPCs. Further studies on the mechanisms mediating neuronal gene transcription are now required to uncover additional factors important for the regulation of neural cell plasticity.

The biological relevance of MeCP2 phosphorylation in AD can be explored using MeCP2 phospho-site-specific knock-in mice that bear mutations in specific bases that blocks phosphorylation. A knock-in mouse model can determine whether MeCP2 phosphorylation or site-specific phosphorylation is an important molecular event required for the regulation of NSCs/NPCs regeneration and plasticity in response to AD damage and if defects in this process are sufficient to explain the neurodegenerative phenotype observed in AD mouse models. It is interesting to assess whether merely a single residue determines the neurological and behavioral phenotypes or whether additive effects of other unknown residues are required. In addition, as both gene transcription and promoter occupancy assessed in this study were limited to a few candidate genes and that MeCP2 acts as a global chromatin regulator, it is likely that additional gene expression changes exist and underlie the neuroprotective effects observed in AD7c-NTP silenced mice.

In summary, we explored the effects of AD7c-NTP silencing as a potential method to promote endogenous NSCs/NPCs regeneration and differentiation following AD injury. This transformation was speculated to be mediated, in part, through the reinforcement of MeCP2 phosphorylation and subsequently a list of lineage-specific gene expressional changes. Notably, we identified a modest increase in neural proliferation in the striatum regions of early AD mice, which was enhanced by AD7c-NTP silencing. These data are consistent with the previously observed upregulation of cell proliferation and neuronal differentiation in the hippocampus and SVZ and partly explains the compensatory hypermetabolism in the cerebral cortex in patients with early AD (Chuang, 2010; Herran et al., 2015; Unger et al., 2016; Wirths, 2017). These findings offer the possibility of replacing degenerated neurons with the most abundant cell types in the mammalian central nervous system, which is a potential therapeutic pathway for cognitive rehabilitation in patients with early AD and slowing the progression of the disease.

## Data Availability Statement

The datasets used and/or analyzed during the current study are available from the corresponding author on reasonable request.

## Ethics Statement

All experiments were approved by our local ethics board in accordance with the National Institute of Health Guide protocols approved by the Medical Experimental Animal Administrative Committee of Tianjin and with the animal protocol approved by the Nankai University.

## Author Contributions

PL and YZ conceived and supervised the study. WQ and ZW designed the experiments. WQ, YC, and HZ performed the experiments. PL and WQ analyzed the data. PL wrote the manuscript. YZ and WQ made manuscript revisions. All authors reviewed the results and approved the final version of the manuscript.

## Conflict of Interest

The authors declare that the research was conducted in the absence of any commercial or financial relationships that could be construed as a potential conflict of interest.
